# *Mycobacterium tuberculosis* Molecular Determinants of Infection, Survival Strategies, and Vulnerable Targets

**DOI:** 10.3390/pathogens7010017

**Published:** 2018-02-01

**Authors:** Davide M. Ferraris, Riccardo Miggiano, Franca Rossi, Menico Rizzi

**Affiliations:** Department of Pharmaceutical Sciences, Università del Piemonte Orientale “A. Avogadro”, Largo Donegani 2, 28100 Novara, Italy; riccardo.miggiano@uniupo.it (R.M.); franca.rossi@uniupo.it (F.R.); menico.rizzi@uniupo.it (M.R.)

**Keywords:** *Mycobacterium tuberculosis*, tuberculosis, antitubercular drugs, target identification, drug discovery

## Abstract

*Mycobacterium tuberculosis* is the causative agent of tuberculosis, an ancient disease which, still today, represents a major threat for the world population. Despite the advances in medicine and the development of effective antitubercular drugs, the cure of tuberculosis involves prolonged therapies which complicate the compliance and monitoring of drug administration and treatment. Moreover, the only available antitubercular vaccine fails to provide an effective shield against adult lung tuberculosis, which is the most prevalent form. Hence, there is a pressing need for effective antitubercular drugs and vaccines. This review highlights recent advances in the study of selected *M. tuberculosis* key molecular determinants of infection and vulnerable targets whose structures could be exploited for the development of new antitubercular agents.

## 1. *Mycobacterium tuberculosis* Pathogenesis

The intracellular pathogen *Mycobacterium tuberculosis* (MTB) is the main cause of human tuberculosis (TB), the ninth cause of death worldwide, and is the leading cause of death from a single infectious agent [1]. According to the last issued WHO Global Tuberculosis Report (2017) [2], an estimated 10.4 million people developed active TB in 2016, and 1.3 million HIV-negative individuals succumbed to the disease, with an additional 374,000 deaths among HIV-positives individuals. An increased threat is represented by multidrug resistant (MDR) and extensively drug-resistant (XDR) MTB strains that are not susceptible to first-line (isoniazid and rifampicin) [3] and second-line injectable drugs (kanamycin, amikacin, capreomycin, and any fluoroquinolone) [4]. Typically, the spontaneous development of such strains is attributed to a poor adherence to the treatment protocols, causing the bacteria to build a resistance toward first-line and second-line drugs.

MTB follows an established route of contagion. MTB bacilli are dispersed in the environment by infected individuals through coughing or sneezing, which cause the bacteria to be scattered in the surroundings. The bacilli, thus aerosolised, are then inadvertently inhaled by nearby individuals, promoting the migration of the bacteria through their respiratory tract and eventually their settling in the host alveoli. There, an early innate immune response occurs, and resident macrophages are recruited to the site of the infection where they phagocyte the infective bacilli, without, however, efficiently clearing the bacterial pool [5]. This process causes a local immune response which culminates in the recruitment of immune cells to the site of infection, leading to the formation of a characteristic infective structure called granuloma which shields the infected macrophages from further attacks by the immune system [6]. Granulomas can preserve the infective bacteria for decades, and the homeostasis between the host’s immune response and the attacking pathogen is disrupted whenever an immunosuppressive condition occurs. Hence, HIV-positive patients are at high risk of developing infective TB, often establishing a direct link between the two pathologies [7].

**Figure 1:**
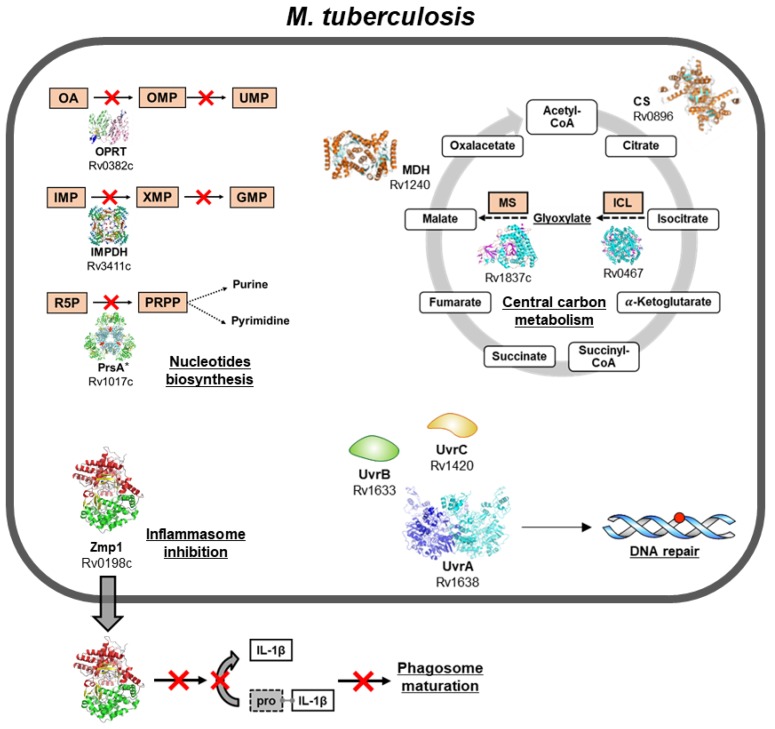
Visual summary, gene names, and structures of the molecular determinants of *Mycobacterium tuberculosis* infection described in the present review. From the upper left to upper right, in a counterclockwise fashion: enzymes involved in the biosynthesis of nucleotides, in inflammasome inhibition, DNA repair, and in the tricarboxylic acid (TCA) cycle, including the glyoxylate shunt. Abbreviations: OA, orotic acid; OPRT, orotate phosphoribosyltransferase; OMP, orotidine 5′-monophosphate; UMP, uridine monophosphate; IMP, inosine monophosphate; IMPDH, inosine 5′-monophosphate dehydrogenase; XMP, xanthosine monophosphate; GMP; guanosine monophosphate; R5P, Ribose 5-phopshate; PRPP, phosphorybosyl pyrophosphate; CS: citrate synthase; MDH: malate dehydrogenase; MS: malate synthase; ICL: isocitrate lyase. * Structure shown is of the *Mycobacterium smegmatis* PrsA homolog [8].

The MTB infection process and its remarkable ability to avoid the attacks of the host’s immune system renders MTB an interesting subject for the study of the host–pathogen interplay. MTB has developed an arsenal of molecular effectors and sophisticated strategies for the success of the infective process. This review offers a panoramic overview of a selection of key molecular determinants of MTB infection and discusses the molecular targets that could be exploited for the development of new antitubercular agents (summarised in Figure 1).

## 2. Innate Immunity and Early Responses to *M. tuberculosis* Infection

Pathogens have evolved elaborate defence and survival strategies for their persistence during infection and replication in a host organism [9]. Likewise, attacked organisms have developed equally elaborate strategies for their protection and the establishment of effective antibacterial defences.

Dendritic cells (DCs) and macrophages constitute the first line of defence against TB and retain complementary roles in the clearance of the infective bacteria [10,11,12]. DCs internalise the infective bacteria and present their antigens to T cells, causing their activation and initiating the onset of the adaptive immune response [13,14,15]; macrophages clear the attacking pathogens by internalising and exposing them to the acidic, hydrolytically active environment of the phagosome, eventually triggering a signalling cascade that culminates in the fusion of the lysosome to the phagosome [16]. Macrophages, together with epithelioid cells and multinucleated giant cells (also known as Langhans’ giant cells) and T lymphocytes are also the principal cellular constituent of granulomas [17,18], the peculiar structures that provides the mycobacterium with a niche in which it can survive and concurrently modulate the immune response over a prolonged period of time [19,20].

The establishment of MTB infection requires a strict control over the production of proinflammatory and anti-inflammatory cytokines. TNF-α, IFN-γ, and IL-1β are particularly important for enhancing the shielding function of the granuloma, whereas IL-10 is one of the main negative regulators of the inflammatory response [21,22,23]. TNFα is a proinflammatory cytokine that promotes granuloma formation, while IFN-γ promotes antigen presentation and the recruitment of CD4+ T lymphocytes and/or cytotoxic T lymphocytes, thereby mediating mycobacterial killing. IL-1β is a proinflammatory cytokine which is mainly produced by monocytes, macrophages, and DCs [24] and is involved in the host’s immune response to Mtb. IL-1β was shown to mediate signals through the IL-1 receptor (IL-1R) in response to mycobacterial infection [25]. Notably, IL-1β is targeted by a MTB secreted protein (Zmp1, Rv0198c) that negatively modulates IL-1β activity, resulting in the quenching of the inflammatory response [26].

In contrast to the pro-inflammatory cytokines listed above, the cytokine IL-10 has anti-inflammatory properties and is produced by macrophages and T-cells upon infection with MTB. IL-10 deactivates macrophage function by downregulating TNFα expression [27], which in turn reduces the production of IFNγ by T-cells and thus aids in Mtb survival.

All these cytokines are secreted and regulated by macrophages and DCs upon detection of specific pathogen-associated molecular patterns (PAMPs) by pattern-recognition receptors (PRRs) which sense MTB signature molecules. The PRRs are responsible for initiating the innate as well as the adaptive immune response to MTB.

## 3. Pathogen Recognition Receptors: How the Host’s Immune System Senses Pathogen-Associated Molecules

Host native immune cells are able to sense the invading pathogens using a plethora of membrane-bound and cytosolic receptors collectively called pattern-recognition receptors (PRRs) [28]. PRRs are responsible not only for detecting specific molecular agonists of bacterial origin, but also for activating the phagocytosis process of the infective bacilli, triggering the signalling pathways that culminate with the production of immunomodulatory cytokines and chemokines [29,30]. Toll-like receptors (TLRs) detect distinct pathogen-associated molecular patterns (PAMPs) derived from viruses, bacteria, mycobacteria, fungi, and parasites, and these include lipoproteins (recognised by TLR1, TLR2, and TLR6), double-stranded (ds) RNA (TLR3), lipopolysaccharide (LPS) (TLR4), flagellin (TLR5), single-stranded (ss) RNA (TLR7 and TLR8), and DNA (TLR9) [31]. Upon recognition of the respective PAMPs, TLRs recruit a specific set of adaptor molecules (such as MyD88) which undergo a homo- and hetero-oligomerisation process leading to the formation of the Myddosome, a multiprotein signalling complex responsible for promoting the proximity of IRAK kinases and their trans-phosphorylation [32]. TLR signalling leads to the secretion of inflammatory cytokines, chemokines, and antimicrobial peptides, resulting in the direct killing of the infectious pathogens [33].

Key TLRs responsible for the detection of intracellular MTB are TLR2 and TLR4, with a dominant role played by TLR2 [34,35]. TLR2 presents a broad substrate specificity for foreign particles, especially for bacterial cell wall components, and is able to sense lipoteichoic acid as well as lipopeptides of fungal and bacterial origin [29]. The broad specificity of TLR2 in ligand recognition suggests that it has a primary role in MTB detection and in the subsequent immune reaction to infection. Indeed, TLR2 has also been linked to autophagy, a key mechanism involved in bacterial clearance [36]. Autophagy is a cellular homeostatic process which is responsible for the clearance of unnecessary or degraded and non-functional cytoplasmic constituents (proteins, lipids, organelles) through the formation of cytoplasmic double-membraned vesicles which eventually deliver the undesired particles either to lysosomes for subsequent degradation or to the cellular membrane for external disposal [37]. The process through which autophagy eliminates bacteria by degradation was first reported in MTB, is called xenophagy [38], and is an essential host defence mechanism responsible for the elimination of bacteria in macrophages and nonphagocytic cells [39,40,41,42].

Complementary to TLR2, TLR4 recognises lipopolysaccharide (LPS) from Gram-negative bacteria, cell wall lipids, glycoproteins, and secreted proteins. Importantly, besides inducing MyD88-mediated signalling like other TLRs, the stimulation of TLR4 can also activate the MyD88-independent TIR-domain containing the adapter-inducing interferon-β (TRIF) pathway, inducing IFN-β secretion [43].

Parallel to TLRs, the nucleotide oligomerisation domain (NOD)-like receptors are cytoplasmic PRRs that are responsible for detecting a number of PAMPs. NOD1 and NOD2, the first identified NLRs, recognise bacterial peptidoglycan moieties [44,45,46] and trigger inflammation by activating NF-κB and MAP kinase (MAPK) pathways [47]. Further study of the NLR family revealed that some members are capable of forming the inflammasome, a multiprotein complex composed by NOD proteins which, once activated upon recognition of pathogen-associated molecules in the extracellular or the intracellular compartment, drives the activation of caspase-1 [48,49], which in turn proteolytically activates pro-IL-1β into IL-1β, the cytokine responsible for the fusion of phagosomes with lysosomes in macrophages and for triggering the early inflammatory response [18], thus promoting bacterial clearance.

In macrophages, the intracellular detection of MTB-infectious molecular patterns is performed by two independent sensing systems that elicit two independent molecular pathways and molecular responses. One molecular pathway involves the inflammasome complex, i.e., NOD-like receptor pyrin domain-containing 3 (NLRP3), or the molecule AIM (absent in melanoma 2) which recognises DNA [50], the adaptor molecule ASC, and Caspase-1, which ultimately is responsible for the maturation of the inactive pro-interleukin 1β (pro-IL1β) into mature IL-1β. The IL-1 family of cytokines, including IL-1β and IL-18, possesses potent proinflammatory activities [51,52,53] and is responsible for the host defence against mycobacteria [54,55].

A second, complementary pathway involved in MTB recognition is responsible for the expression of type I interferons following the activation of the PRR STING sensor protein which senses cyclic dinucelotides [56]. DNA sensing can also be accomplished by the protein cGAS (cyclic GMP-AMP synthase (cGAS), which sequentially triggers the second messenger cGAMP which engages STING as a secondary receptor [57].

Notably, these two independent detection systems are redundant, and the knockout of single PRRs molecules does not inhibit the production of proinflammatory cytokines and chemokines [58]. However, the inhibition of the central molecules involved in PRR signalling induces lethality in mice models [59,60,61]. Moreover, it has been observed that TLR and NLR signalling pathways are shared by the autophagy signalling, suggesting a crosstalk between innate immunity and autophagy in clearing MTB infection [62].

## 4. *M. tuberculosis* Subversion of the Host’s Immune System: The Case of Zmp1

A key step for the successful bacterial removal by macrophages is the phagosome maturation process, which leads to the formation of the phagolysosomes and the full clearance of the invading pathogens [10]. MTB have evolved the ability to prevent their killing by macrophages via inhibiting the phagosome maturation process [5,63]. This step is critical for the progression of the infection, since it compromises bacterial clearance [64] and antigen processing [65,66]. A seminal paper by Master et al. [26] proposed that the secreted protein Zmp1 (Rv0198c) is involved in the suppression of the inflammasome activation by inhibiting caspase-1, eventually preventing the processing of pro-IL-1β into IL-1β and the consequent phagosome maturation. The authors showed that the *zmp1* mutants bacteria were localised in the phagosome, which could also undergo maturation into phagolysosomes, and that inhibition of caspase-1 could reverse this phenotype. Moreover, the suppression of *zmp1* restored the activation of caspase-1, the production of IL-1β, and the full maturation of the phagosome into a phagolysosome, leading to the clearance of the pathogen. In addition, they showed that exogenously added IL-1β was sufficient to determine bacteria clearance by the phagolysosome. Hence, the authors proposed a direct link between Zmp1 expression and the inhibition of inflammasome activation.

Zmp1 is a Zn-dependent metalloprotease which shares 31% homology and 48% similarity with both human peptidase neprilysin (NEP) and human endothelin-converting enzyme-1 (ECE-1), and the Zmp1 crystal structure revealed key residues that could be exploited for the design and development of specific inhibitors, especially regarding the nonconserved Arg615 and Arg616 [67]. Although the molecular partner(s) of Zmp1 has not been characterised, efforts have been made to identify Zmp1 substrate sequence specificity [68] and in the engineering and development of inhibitors against Zmp1 activity [69,70].

In light of the newly discovered function of Zmp1, recent studies have aimed at the development of novel anti-MTB vaccines based on *zmp1*-mutant strains. Notably, a Bacillus Calmette–Guérin (BCG) *zmp1* mutant exhibited increased antigen presentation [71], while preclinical studies showed that BCG *zmp1* mutants are more immunogenic and as safe as BCG strains. Hence, BCG *zmp1* mutant strains can be considered promising candidates for the development of a novel anti-TB vaccine, and clinical testing has been considered [72].

## 5. Advances in Molecular Target Identification for Tuberculosis Drug Discovery

Most of the used antitubercular drugs target essential enzymes involved in vital cellular biological processes such as cell wall synthesis, energy metabolism, protein synthesis, phosphate transport, and the metabolism of key molecules and cofactors [73,74,75,76,77].

Several efforts and a variety of experimental approaches have been utilised for the identification of essential MTB genes [78,79,80,81], and phenotype screening is emerging as a powerful technique in the search for phenotype-associated genes and for the screening of libraries of compounds active against MTB [82]. This approach, once neglected in favour of target-based screening, is facing a significant resurgence due to the technological advancements concerning high-throughput genetic and big data analyses. The phenotypic screening allows a direct and measurable phenotypic response of all MTB cells against a library of compounds, measuring and evaluating the efficacy of such molecules in bacterial killing and/or alteration of their general viability. This technique circumvents the problematics related to the upstream identification of target essentiality and drug-associated complications such as drug permeability and metabolism. Advances in high-throughput automations, genome sequencing, chemical biology, and data handling have dramatically advanced the phenotypic screening technique and expanded its applications, allowing the discovery of new anti-infective compounds and new vulnerable targets. The practical success of the phenotypic screening is evidenced by the development of new antitubercular drugs, some of them currently under study [83]. Overall, these researches have highlighted the importance of molecules essential to the MTB metabolism (and of the enzymes involved in their biosynthesis) for the development of novel antitubercular drugs.

We highlight here the recent structural reports of some of the essential enzymes involved in the synthesis of key metabolites necessary for MTB survival that could constitute promising targets for the development of new antitubercular compounds.

## 6. Enzymes of the Nucleotide Biosynthesis Pathways as Targets for the Development of New Antitubercular Molecules

The de novo and salvage syntheses of purine and pyrimidine nucleotides, the key precursors of DNA and RNA, have been reported as essential for mycobacterial survival [84] and therefore represents a source of promising targets for the development of new antitubercular drugs. Recent publications report the structural characterisation of key enzymes involved in the synthesis of essential molecules for the purine and pyrimidin base biosynthesis. In vitro and in vivo analysis showed that de novo and salvage syntheses of purine and pyrimidine nucleotides are essential for mycobacterial survival, thus representing a source of promising drug targets against TB [84].

The pyrimidine synthesis pathways converge on the formation of the key molecule uridine 5′-monophosphate (UMP), the precursor of all pyrimidine nucleotides [85]. A key component of its biochemical synthesis is the orotate phosphoribosyltransferase enzyme (OPRT, *pyrE*, Rv0382c) which converts orotic acid into orotate monophosphate. The crystal structure of OPRT revealed an unprecedented and serendipitous presence in the active site of a metallorganic molecule, originated from the crystallisation reservoir, which makes specific interactions with the protein [86]. Organometallic compounds represent a new class of drugs that holds promise for the development of novel antibacterial agents with limited antimicrobial resistance [87]. The study of new organometallic molecular scaffolds for antimycobacterial drug development could represent a new research area for the design of new, active molecules [88]. In the context of TB, where antimicrobial resistance can lead to the onset of MDR and XDR MTB strains, the research and development of new organometallic inhibitory molecules offers unprecedented opportunities for the development of new antitubercular drugs.

MTB has the ability to either synthesise purine nucleotides de novo or to scavenge them from the host [84,89]. The de novo biosynthetic pathway for guanine-containing nucleotides as well as the salvage pathways of purine nucleotides converge on the synthesis of a common intermediate, inosine 5′-monophosphate, from which the required guanine- and adenine-containing deoxynucleotides, precursors for DNA synthesis, are derived. In particular, guanine-containing precursors require the conversion of inosine 5′-monophosphate to xanthine 5′-monophosphate through the action of inosine 5′-monophosphate dehydrogenase (IMPDH). Among the three MTB IMPDH homologs (*guaB1*, *guaB2*, and *guaB3*), only one (*guaB2*, Rv3411c) has been shown to be essential for the NAD^+^-dependent dehydrogenation and hydrolysis of inosine 5′-monophosphate to xanthine 5′-monophosphate [90,91]. In the context of phenotypic screening, a promising molecule (VCC234718) could induce MTB mortality in macrophages and mouse lung xenografts, while retaining low toxicity in mammalian cells [92,93]. Notably, the resistant MTB strain developed a spontaneous mutation (Y487C) in the *guaB2* gene, thus identifying a key mutation conferring MTB resistance to the active molecule. However, several efforts failed to determine the crystal structure of MTB IMPDH. An alternative strategy for the elucidation of the IMPDH structure consisted in the crystallisation of the *Mycobacterium thermoresistible* IMPDH homolog, which instead readily crystallised. The structure revealed the molecular determinants of the failed recognition of the active compound by the IMPDH Y487C mutant and structurally explained the uncompetitive inhibition suggested by the biochemical assays. Hence, this seminal work proved IMPDH as a vulnerable target for MTB drug development, evidenced the structural determinants of bacterial resistance towards the inhibitor, and further suggested the crystallisation of homologous target proteins as a valid alternative strategy for dissecting the molecular determinants of bacterial resistance to anti-infectives [92].

An analogous approach has been devised for the structural elucidation of the PrsA enzyme (Rv1017c), the sole responsible enzyme for the Mg^2+^-dependent conversion of ribose 5-phosphate (R5P) to phosphoribosyl pyrophosphate (PRPP) using ATP [94]. PRPP is a key metabolite involved in several biosynthetic pathways including those for histidine, tryptophan, nucleotides, and decaprenylphosphoryl-arabinose, an essential precursor for the mycobacterial cell wall components [95,96]. In addition, PrsA is upstream of DprE1 in this pathway [97], and DprE1 is a preclinically validated target, with multiple inhibitors under development [98]. PrsA has been proved to be essential for *M*. *tuberculosis* survival and multiplication in vitro, and conditional knockout mutant strains showed decreased viability and alteration in bacterial morphology [97]. Hence, PrsA is a critical enzyme for MTB metabolism and cell survival.

Efforts have been devolved for the crystallisation of MTB PrsA, however without success. As for the IMPDH protein illustrated above, an alternative strategy has been developed, and the PrsA homolog from *Mycobacterium smegmatis* was preferred, leading to its crystallisation and structure solution, which, thanks to the high (87%) identity with its MTB homolog, could serve as a valid model for the engineering and development of new antitubercular molecules [8].

## 7. Enzymes of the Tricarboxylic Acid Cycle as Drug Targets

The central carbon metabolism (or tricarboxylic acid, TCA, or Krebs cycle) is of paramount importance for all aerobic bacteria. The TCA cycle is a complex, highly regulated converging hub of a series of chemical reactions that provide, with their proper functioning, a vast array of metabolites and molecules necessary for the survival and homeostasis of any aerobic organism. The central carbon metabolism provides important molecules and nutrients to the cell by consuming the breakdown products of carbohydrates and fatty acids and by producing essential intermediate molecules, reducing agents, and ATP needed for the normal functioning of the cell. The TCA cycle is also of paramount importance for the adaptation of the pathogens to the host environment [99], and the enzymes involved in the cycle constitute vulnerable drug targets. However, the targeting of these enzymes poses significant challenges because of their sequence similarities with their human homologs and of their high evolutionary conservation, making it highly challenging to identify specific inhibitory molecules for the MTB enzymes. Notably, however, Kasbekar et al. [100] reported the discovery of a specific inhibitor of MTB fumarate hydratase which binds to an allosteric site and interacts with nonconserved residues between the human and the MTB homologs. This work encourages and demonstrates the feasibility of the discovery of specific inhibitors targeting highly evolutionary conserved enzymes in MTB and the host, with significant implications for future antitubercular drug developments.

Attractive targets for the MTB TCA cycle are also represented by the enzymes involved in the “glyoxylate shunt”, an alternative route of the TCA cycle that bypasses those steps that lead to a loss of CO_2_. In this bypass, two enzymes are involved, namely the isocitrate liase (ICL, Rv0467) and the malate synthase (MS, Rv1837c), and an earlier study showed that the inhibition of ICL is fatal for MTB [101]. Since the elucidation of the crystal structure of ICL in 2000 [102], efforts have been devolved in the research of effective molecules targeting the enzymes of the glyoxylate shunt [103]. Moreover, pharmaceutical companies and international alliances invested considerable resources in the intense screening of library molecules, but with limited success [82,83,84]. Hence, the TCA cycle still remains a promising target for extracellular MTB, and recent efforts have been made to elucidate the structures of MTB Krebs cycle enzymes that could be useful for the design of specific inhibitors [104].

## 8. *M. tuberculosis* DNA Repair System as a Drug Target

One of the hallmarks of MTB infection is its ability to survive the hostile environment of the host macrophages, where a series of multiple environmental stresses (i.e., highly acidic conditions, oxidative and nitrosative stresses, oxygen deprivation, nutrient depletion) challenge the survival of MTB for a prolonged period, even for decades. Moreover, each stage of the complex MTB life cycle is characterised by the continuous exposure to DNA-damaging stresses that could compromise bacterial fitness as a result of genomic instability [105]. By focusing on the long-term persistence inside infected macrophages, MTB must deal with endogenous DNA-alkylating chemical species originated by the action of highly reactive oxidative (ROS) and nitrosative (RNI) radicals [84,106,107]. Since DNA stability favours the infection process, while DNA damage critically impairs it [105], MTB have evolved DNA-repairing strategies to preserve their genome stability and bacterial survival during infection.

Extensive MTB genomic analyses, together with a more limited number of gene inactivation studies, have identified components of most of the DNA repair pathways which are active in other species, including multi-enzymatic systems like Nucleotide Excision Repair (NER), Base Excision Repair (BER), and recombination repair systems—with the exception of canonical Mismatch Repair (MMR) components—as well as proteins responsible for Direct Reversal of DNA damage [108]. Notably, a NucS-dependent DNA repair system that potentially replaces the MutS/MutL-based MMR was recently identified in *M. smegmatis* [109].

Proteins involved in DNA metabolism could be considered as drug targets since many of them provide essential functions to the bacteria and, in many cases, belong to biochemical pathways that are distinct from the human ones at the biochemical and structural level. Starting from the establishment of the primary infection until the post-latency reactivation, ROS and RNI represent the most significant sources of DNA damage along the whole MTB infection cycle.

Several studies have confirmed the role of mycobacterial proteins involved in NER and BER in the removal of DNA products that arise as a consequence of the exposure to ROS and RNI. Interestingly, the MTB genes *ada/alkA* and *ogt* are upregulated in both human [110] and murine macrophages [111,112] and they are also upregulated in response to oxidative stress in the broth culture model [111,113]. The product of the *ogt* gene in MTB is a key protein involved in the repair of alkylated DNA, namely, O^6^-methyl-guanine methyl-transferase (OGT; Rv1316c), which recognises the promutagenic O^6^-methylated guanine found in alkyl-damaged DNA. OGT irreversibly transfers the damage-associated O^6^-alkyl group from the modified guanine to a conserved cysteine in the protein active site, which is hosted in the C-terminal domain of the protein, promoting also its degradation [114]. In vitro and in vivo studies using a mouse model showed that OGT is not essential for MTB infectivity and survival [78,106,115,116]. However, it has been observed that OGT is important for protecting the mycobacterial DNA at different stages of the infection and that OGT expression undergoes a fine-tuning regulation during the infection in response to alkylating agents [111,113,115]. Two recent publications report the biochemical and structural characterization of the protein in its ligand-free form and in complex with modified DNA [114,117]. Interestingly, two point-mutated variants of the protein, mimicking the ones occurring in multidrug resistance MTB clinical isolates, were biochemically characterised, highlighting, although to different extents, their reduced DNA-binding affinity that could contribute in vivo to a “hypermutator” phenotype, possibly beneficial to the fitness of the bacilli under circumstances of selective pressure [114].

Concerning the most important multienzymatic response to oxidative and alkylating stresses, i.e., NER, the first steps of this DNA repair cascade are well characterised and they are carried out by the coordinated action of the UvrA, UvrB, and UvrC proteins [118]. Recent research articles have provided an in-depth biochemical and structural characterisation of the UvrA and UvrB proteins that act in the first step of the NER pathway [119,120,121], demonstrating that UvrA proteins interact with UvrB in the absence of ligands, and suggesting this protein–protein interaction as a potential drug target for blocking the entire NER cascade in MTB. Moreover, an inhibitor of the endonuclease activity of the UvrABC complex as a whole was identified [2-(5-amino-1,3,4-thiadiazol-2-ylbenzo[f]chromen-3-one) (ATBC)], and it resulted to be active at a micromolar concentration. However, the molecular basis of ATBC activity and the direct target inside the UvrABC system are still unknown [122].

Considering that the interdiction of different MTB responses to oxidative stresses, which are constantly present in its life cycle, could result in genomic instability and in an altered bacterial fitness, the concomitant inhibition of NER- and OGT-dependent direct DNA reversal pathways could be considered as a pharmacological strategy for potential antitubercular treatments.

## 9. Conclusions

Although human TB can be traced back to 70,000 years ago [123], today it still remains one of the deadliest human infectious diseases, further exacerbated by the emergence of MDR, XDR, and totally drug-resistant (TDR) TB as well as of HIV coinfection with TB. The resilience of such resistant strains to antitubercular treatments has been so severe that nowadays surgery is being again used as a supplement to drugs for the treatment of TB, and in 2016 the WHO made recommendations about the use of surgery for patients with MDR-TB [124]. Moreover, the only available antitubercular vaccine is the *Mycobacterium bovis* BCG vaccine which was used in humans in the 1920s [125] and is still used today, although it does not protect infants completely against TB and is unreliable against adult pulmonary TB [126]. Hence, there is a pressing need for the research of new, effective antitubercular drugs and vaccines.

Despite the great potential of the target-based screening for the discovery of new antitubercular molecules, this approach has been mostly unsuccessful in tuberculosis drug research because of the uncertainty and unpredictability of the molecules’ absorption and general biology. On the contrary, whole cell phenotypic screening gave rise to a remarkable success rate of active molecules. Prominent examples of the feat of such an approach are the discovery of bedaquiline by Andries et al. [127] through whole-cell screening assays using diarylquinoline molecules and the discovery of the anti-TB properties of the natural compound griselimicyn of *Streptomyces* origin [128]. However, recent research has reported the successful combination of the target-based and phenotypic screening approach using knockout mutant strains and reporter genes for the identification of new anti-TB compounds [129]. This combined approach could lead to the development of new anti-TB molecules with multiple modes of action, which is highly beneficial for TB cure since tuberculosis therapy requires the use of multiple drugs, each with different modes of action.

As alternatives to such resource-intensive drug discovery strategies, structural biology and in silico screening of molecules can contribute to the development of new antitubercular agents and could be envisaged as promising, less demanding strategies for the screening of very large compound libraries [130,131]. Such an approach led to the identification of inhibitors of essential drug targets such as DprE1 and InhA; however, the validation of their antitubercular properties still await a definitive answer [129]. 

Drug repurposing also represents a promising approach for the research and development of antitubercular agents, that aims at mitigating the intrinsic costs and the lengthy times necessary for the approval of a new drug. Repurposed drugs already possess pharmacokinetic and safety data, which are beneficial during the approval process. Compound families such as oxazolidinones, riminophenazines, rifamycins, and fluoroquinolones are all examples of repurposed drugs that were used for the treatment of other diseases and that are currently being evaluated for TB treatment [129].

The research and development of new antitubercular drugs and treatments is still a demanding, resource-consuming, and lengthy path that sees the cooperation of international players, governments, and supranational unions and the coordinated efforts of academic, public and private organisations. The TB-related molecular mechanisms and protein targets here reviewed offer a structural perspective on essential and important pathways and molecular targets that could be exploited for the development of new, effective anti-TB agents.
